# Contexts of cigarette and e-cigarette use among dual users: a qualitative study

**DOI:** 10.1186/s12889-015-2198-z

**Published:** 2015-09-04

**Authors:** Pallav Pokhrel, Thaddeus A. Herzog, Nicholas Muranaka, Sakshi Regmi, Pebbles Fagan

**Affiliations:** Cancer Prevention & Control Program, University of Hawaii Cancer Center, 701 Ilalo St, Honolulu, HI 96822 USA; School of Public Health and Health Sciences, University of Massachusetts Amherst, 715 North Pleasant Street, Amherst, MA 01003 USA

## Abstract

**Background:**

Not much is currently understood regarding the contexts of cigarette and e-cigarette use among dual users. Proper application of e-cigarettes to smoking cessation or tobacco harm reduction would require an understanding of when and why dual users use cigarettes versus e-cigarettes. This study sought to elucidate the contexts of cigarette versus e-cigarette use among dual users.

**Methods:**

Twelve focus group discussions were conducted with 62 young adult current daily e-cigarette users [63 % men; mean age = 25.1 (Standard Deviation = 5.5)]. Almost all participants either concurrently smoked cigarettes or had been recent dual users. Data were analyzed following principles of inductive deduction.

**Results:**

Results indicated that dual users’ use of cigarettes is influenced by particular activities (e.g., before/after eating), strong craving or need for stimulation (e.g., in response to stress), places/situations (e.g., when cigarette smokers are nearby; outdoors), use of other substances (alcohol, coffee), and unavailability of an e-cigarette when needed. In addition to particular activities and places/situations that are conducive to e-cigarette use, use of e-cigarette when cigarette is not available or where cigarette smoking is not permitted emerged as contexts specific to e-cigarette use.

**Conclusions:**

For habitual cigarette smokers wanting to quit tobacco smoking, switching over completely to e-cigarettes may require skills of cognitive-behavioral management. Future research needs to ascertain the characteristics of dual users who use e-cigarettes as cessation aids versus as cigarette alternative when cigarette is unavailable or smoking is not permitted.

## Background

Electronic or e-cigarettes are battery-powered devices that deliver vapor which may be inhaled in the manner tobacco is smoked. The vapor is released when a liquid—popularly known as e-liquid or e-juice—is heated. The e-liquid is usually a solution containing propylene glycol and/or vegetable glycerin, nicotine, and flavor concentrates. Ready consensus has been lacking regarding the long- and short-term public health consequences of e-cigarette use, including the effects of e-cigarette use on tobacco use initiation, maintenance or cessation [1]. However, it is generally believed that e-cigarettes deliver fewer toxins and carcinogens into the human body compared with cigarettes and thus have potential for application in tobacco smoking cessation or harm reduction efforts [2]. Recent reports indicate that e-cigarette use prevalence rates among U.S. and U.K. adults are steadily increasing over the past few years [3–5]. For example, among U.S. adults, prevalence of ever e-cigarette use increased from 1.8 % in 2010 to 13 % in 2013 [3, 5].

Of the U.S. adult current daily and intermittent cigarette smokers, 30 and 34 %, respectively, are likely to report current daily or occasional e-cigarette use [5]. It is not clear what proportion of cigarette smokers who try e-cigarettes actually switch over completely to e-cigarettes, reject e-cigarettes or continue to use both cigarettes and e-cigarettes (i.e., dual use). It appears that relatively fewer adult cigarette smokers who try e-cigarettes persistently use e-cigarettes daily for a substantive length of time (e.g., a month); the majority are likely to either reject e-cigarettes or become intermittent users [6]. Further, only a few daily e-cigarette users among adult cigarette smokers (e.g., 20 %) [6] may be able to abstain from smoking cigarettes completely. The majority of current cigarette smokers who use e-cigarettes are likely to be dual users, although there is no clarity as to for how long.

An obvious problem with the concomitant use of cigarettes and e-cigarettes is that such dual use may help perpetuate tobacco smoking habit [7]. There are many ways in which e-cigarettes may make the life of a cigarette smoker easier. For example, because e-cigarette vapor lacks the noticeable smell of tobacco, e-cigarettes may be surreptitiously used in places where smoking is not allowed. For the same reason, smokers who would not smoke cigarette inside their homes may use e-cigarette indoors, thus overcoming the need to step outdoors to smoke. Lower perceived harm associated with e-cigarette vapor and second-hand exposure to the vapor may further promote the use of e-cigarettes indoors, including at home and work-place. Thus by reducing inconveniences and providing a means to deal with craving or withdrawal symptoms when cigarette smoking is not possible, e-cigarettes may help smokers maintain their cigarette smoking behavior.

Conversely, habitual cigarette use may be perceived as a hurdle to smoking cessation among daily e-cigarette users who are using e-cigarettes to quit smoking. Smoking cessation or reduction is the most common reason for e-cigarette use among adult cigarette smokers [8, 9]. However, research indicates that only smokers who are older, more educated, and highly motivated to quit smoking are able to completely replace cigarette smoking with e-cigarette use [6, 10]. Thus far, very little has been known about the contextual factors that contribute to continued use of cigarette use among daily e-cigarette users. For example, knowledge is limited regarding why and when daily e-cigarette users use cigarettes. Such knowledge is important to effectively employ e-cigarettes in smoking cessation or tobacco harm reduction efforts. Understanding the contextual factors that may function as barriers in smoking cessation/reduction will help develop strategies that may be used as adjuncts to e-cigarettes in smoking cessation or harm reduction interventions. This study was conducted to gain an in-depth understanding of the contextual factors of cigarette use through focus group discussions with current daily e-cigarette users the majority of whom are likely to be past or current cigarette smokers. In addition, we sought to compare the contexts of cigarette versus e-cigarette use.

## Methods

### Procedures

#### Recruitment

Participants were recruited in Hawaii, mainly Oahu, through advertisements in print media (e.g., newspapers, magazine) and through distribution of flyers on college campuses. The study was advertised as a focus group study with young adult (18–35 year old) e-cigarette users. The sample was restricted to young adults because of the increased vulnerability of young adults to both cigarette and e-cigarette use [4, 11]. Interested individuals telephoned the research site and were screened by a research staff. They were invited to participate in a focus group session if they met the study inclusion/exclusion criteria. Participants were required to be 1) current daily e-cigarette users; 2) between 18 and 35 years in age; 3) fluent in English; and 4) able and willing to travel to the research site. Verbal consent was obtained from potential participants before the screening was conducted. Five to six selected individuals were invited to participate in one focus group session. Participants whose friends or family members had also been selected to participate in the study were invited to participate in separate groups. Because the discussions focused on a gender-neutral topic such as e-cigarettes, groups convened were mixed-gender. Attempts were made to balance the number of men and women in a group.

This study was approved by the Western Institutional Review Board (WIRB). Participants were asked to visit the research site with a government-issued ID and their electronic cigarette product of regular use. Once the age was verified, participants read the informed consent form, provided written consent and completed a self-report questionnaire which included measures of demographic characteristics (i.e., age, gender, ethnicity, and annual household income), and e-cigarette use and cigarette use behavior. Participants were explained during the consent procedure that participation was voluntary and all information they provided would be kept confidential and their private, identifying information would not be linked with the data they would provide. Focus group discussions commenced immediately after all group members had completed the self-report questionnaire. None of the interested potential participants who met the inclusion/exclusion criteria declined to participate in the study.

#### Focus groups

Focus group discussion was selected as a research methodology for this study because focus groups offer an efficient way to explore and discover wide-ranging information on a topic through discussions with individuals whose experience with the subject-matter being studied is first-hand. Focus groups have an advantage over individual interviews in that interactions among participants in a group setting add both depth and breadth to the data. In the current study, total 62 individuals participated across 12 focus groups over a period of 4 months, from November 2013 to February 2014. On average, each focus group consisted of 5 individuals and each discussion session lasted for approximately 1 h and 15 min. The same protocol was followed across all sessions. After the initial “engagement” questions (e.g., “How did you first come to know about e-cigarettes?” “What were your first impressions of e-cigarettes?”), participants were asked three sets of questions, of which only one set concerns the present analysis. The main question was: “If you currently smoke cigarettes, when do you smoke cigarettes and when do you use e-cigarette?” The same question was altered to address those who claimed to have quit smoking cigarettes: “At the time when you were using both cigarettes and e-cigarettes, when did you use cigarettes and when did you use e-cigarettes?” Participants were asked to provide contexts as well as the reasons for dual use.

All focus groups were moderated by the first author. The moderator was accompanied by a note-taker (third-author) in all sessions. The same person functioned as a note-taker across all sessions. The note-taker noted each participant’s contribution to the discussions as well as his or her non-verbal behavior. All sessions were audio-recorded. The note-taker transcribed the audio-recorded data following each session. The transcriptions were cross-checked against the audio-recording and focus-group notes by the moderator. Identities of subjects and places or events that might identify subjects were excluded from transcripts. Next, the moderator and the note-taker reviewed transcriptions, notes and discussed areas of consensus and discrepancies. Discrepancies were discussed with the second and fourth authors, who did not participate in moderation or data collection. The second and fifth authors reviewed the transcriptions and reached conclusions regarding discrepancies. Transcriptions were considered to be ready for analysis after discrepancies were addressed. Then the data were coded separately by the moderator and the note-taker. The other members of the research team independently reviewed the codes and coding procedures. The research team met to review the coding and coding procedures and address discrepancies. Recruitment was stopped after the 12th session because 11th and 12th group discussions added no new information, thus implying data saturation.

### Data analysis

Quantitative data were analyzed by using SAS (Version 9.3) software. The NVIVO software (Version 9) was used to code, manage, and analyze the qualitative data. Data were analyzed following the principles of inductive content analysis [12]. First, open coding was performed. The moderator and the note-taker separately read the transcripts, noting down each context related to cigarette or e-cigarette use. At this stage, the maximum possible number of context categories was generated separately for cigarettes and e-cigarettes. Thus a list of codes was created on an ongoing basis. Next, the codes were grouped under higher order concepts. We looked for cigarette/e-cigarette use contexts across focus groups and identified convergent and divergent contexts. A final master code list was created along with primary or higher order code definitions up to the point of saturation [13, 14]. We compared and contrasted the codes for all 12 focus groups. Because our focus groups were not stratified by gender or race/ethnicity, we report the data for the multiethnic, mixed-gender groups used to collect the data.

## Results

### Participants

Table 1 summarizes participants’ demographic and e-cigarette and cigarette use characteristics. Participants ranged between 18 and 35 years in age. More men were represented in the sample than women and the sample was ethnically diverse with approximately 55 % representing Asian/Pacific Islanders. Forty one percent of the participants had been using e-cigarettes for 1 year and more and 41 % had been using e-cigarettes for 2–6 months. Approximately 26 % of the participants were light e-cigarette users (i.e., vaped only few times a day) and 56 % were heavy e-cigarette users (i.e., vaped frequently or constantly throughout the day). Forty-eight percent of the participants smoked cigarettes daily and 36 % smoked cigarettes occasionally. Thus, 84 % of the participants were current dual users of cigarettes and e-cigarettes. Of the *n* = 10 participants who identified themselves as current non-smokers, two participants had never smoked cigarettes whereas eight participants were former cigarette smokers. The former smokers reported having smoked >100 cigarettes in the lifetime and none in the past 30 days. In summary, 97 % of the current sample represented current or former dual users.

**Table 1:** Participant characteristics (*N* = 62)

Characteristics	Mean (SD)/Frequency (n)
Age	25.1 (5.5)
Gender	
Male	63 % (39)
Female	37 % (23)
Ethnicity	
Asian-American	19 % (12)
African-American	8 % (5)
Filipino	12 % (8)
Hispanic/Latino	7 % (4)
Native Hawaiian/other Pacific Islander	23 % (14)
White	31 % (19)
Annual Income	
0–$19,999	30 % (18)
$20,000–$29,999	16 % (10)
$30,000–$39,999	13 % (8)
$40,000–$49,999	20 % (12)
$50,000 or more	21 % (13)
Length of time having been using e-cigarettes	
Less than a week	0 % (0)
About 1–2 weeks	2 % (1)
About 1 month	12 % (7)
About 2–5 months	26 % (16)
About 6 months	15 % (9)
About 7–11 months	5 % (3)
About 1 year	21 % (13)
More than a year	20 % (12)
Size of e-liquid container usually bought	
15 ml	68 % (39)
30 ml	32 % (18)
Other	0 % (0)
Length of time a container lasts	
More than a month	23 % (14)
About a month	16 % (10)
3–4 weeks	10 % (6)
1–2 weeks	38 % (23)
Less than a week	8 % (5)
2–3 days	3 % (2)
1 day	2 % (1)
Less than a day	0 % (0)
Daily e-cigarette use behavior	
Vape only few times a day	26 % (16)
Vape frequently but only at certain times of the day	18 % (11)
Vape frequently throughout the day	40 % (25)
Vape constantly throughout the day	16 % (10)
Lifetime cigarette use	
Never smoked a cigarette	3 % (2)
Smoked < 100 cigarettes	18 % (11)
Smoked ≥ 100 cigarettes	79 % (49)
Cigarette use behavior	
Do not smoke	16 % (10)
Smoke sometimes	36 % (22)
Smoke daily	48 % (30)
Past-30-day cigarette use	
Yes	63 % (39)
No	37 % (23)

Note. % values were rounded to the nearest whole number

### Contexts of cigarette use

Table 2 lists the contexts of cigarette use that emerged across the 12 focus groups, supported by quotes that all authors agreed best exemplified the corresponding context. The contexts could be classified into five primary categories: strong craving/need for stimulation, activities, places/situations, and use of other substances that cue or encourage cigarette use, and use of cigarettes as e-cigarette substitutes. Each of these categories and the contexts subsumed within them are described below.

**Table 2:** Contexts of cigarette use

Contexts	Quotes
Craving/stimulation	
When strong stimulation is needed	“And I just recently bought a pack of cigarettes, over the weekend, just because I had to go to work at 5:00, and I only got two hours of sleep that night, so I was really off, like super tired. So I went to work, and at my break, I went to buy a pack of cigarettes to wake me up even more, so I could deal with customers and not seem tired. Because it’s so immediate, the effect of nicotine.” (20 year old man)
When craving is strong	“It’s kind of hard to quit cigarettes though, it gives you that craving and you absolutely need it.” (23 year old woman)
When stressed	“So there are times when I’m like, stressed out, or even if I’m just bored or had a long day or will have a long day like today, I’ll definitely go out for a cigarette.” (28 year old woman)
As laxative	“So in the morning if I have to poop, I need a cigarette. Because I need to relax my body to get stuff out.” (25 year old man)
Activities	
After/before eating	‘I think after you eat and you’re really full, cigarettes are better. Like for me I like it a lot better than my e-cig.’ (30 year old man)
After work-out	“Actually my favorite time to smoke cigarette is after a workout. Because you just worked a lot of it out of your system, and then it hits you with the best head buzz, like you just started it again.” (24 year old man)
After waking up/before going to bed	“[I have] been vaping for six months. I still smoke cigarettes twice a day. Once in the morning after waking up and once in the evening before going to bed”. (31 year old woman)
After sex	“After a meal or after sex the e-cigarettes don’t cut it, you know what I mean?” (33 year old man)
Places/situations	
When cigarette or smokers are nearby	“And it depends on if cigarettes are near you. Like you said your roommate smokes, and my husband, when he’s home, he smokes cigarettes. So if there are cigarettes in my house, and they’re right there, it’s harder than if I just don’t buy them.” (33 year old woman)
Special occasions	“So I’m a professional musician, so after gigs, usually. And then at rehearsals. Yeah, can’t avoid it.” (32 year old man)
When not scheduled to meet anyone	“If I don’t have to see anybody for a few hours I’m going to smoke a regular cigarette.” (26 year old female)
Outdoors	“I joke around it’s like I use e-cig to smoke indoors and use cigarettes to smoke outdoors.” (35 year old man)
On the weekend/While partying	“I switch over to cigarettes on the weekend, when I am partying.” (22 year old man)
When socializing with friends	“Other people smoking. Like if you’re with a group, and people go, ‘Let’s go outside for a smoke.’ Then you want to. It’s hard to be like, ‘I'll go with you, with my e-cig.’ I mean you can, which is nice, but it’s easy to want a real cigarette in that situation.” (26 year old woman)
Other substances	
When drunk	“When I’m drunk, I do prefer smoking cigarettes.”(27 year old man)
When using marijuana	“But also the weed factor plays into it, because then I also need a real cigarette.” (22 year old man)
With coffee	“I usually have to have a cigarette in the morning, when I’m drinking coffee.” (33 year old man)
E-cigarette substitute	
When e-cigarette is not available	“Like I said, I don’t have that urge to smoke. The only time I will smoke a cigarette is if I go out drinking, and this will die on me. Then I go out and buy a pack, or if I want to have a cigarette I’ll have a cigarette. But then I’m fine, I don’t feel the urge to keep going.” (30 year old man)
When switching from one e-cigarette to another	“When I first started I quit smoking cigarettes for a good three or four months, I didn’t have a single cigarette. And then I don’t know what happened, I had the little one like that [e-cigarette], but it just wasn’t doing it for me, it wasn’t strong enough. So I switched to the bigger one. But in that time period before I switched to the bigger one, I started smoking cigarettes again.” (33 year old woman)

#### Craving/stimulation

This category includes contexts of cigarette use that are characterized by strong craving or need for stimulation. Four specific contexts were found: use of cigarettes 1) when strong stimulation is needed (10 groups); 2) when craving is strong (10 groups); 3) when stress is high (9 groups); and 4) as laxative (6 groups).

#### Activities

Four contexts of cigarette use emerged that may be broadly classified as activities that cue or encourage cigarette smoking: 1) after/before eating a meal (5 groups); 2) after work-out (2 groups); 3) after waking up/before going to bed (4 groups); and 4) after sex (6 groups).

#### Places/situations

Participants mentioned that they chose to, or were influenced to, smoke cigarettes depending on places and situations. Seven contexts emerged. Dual users reported that they were more likely to smoke cigarettes 1) when cigarette smokers are close by (8 groups); 2) when socializing with friends (8 groups); 3) on the weekend/while partying (9 groups); 4) on special occasions (4 groups); 5) when not scheduled to meet or see anyone (2 group); 6) when outdoors (7 groups).

#### Other substances

Participants mentioned that cigarette smoking, compared with e-cigarette use, was preferred while consuming other drugs or psychoactive substances, specifically: 1) alcohol (11 groups); 2) marijuana (2 groups); and 3) caffeine (3 groups).

#### E-cigarette substitute

Some dual users mentioned that they smoked cigarettes only 1) when an e-cigarette was unavailable (9 groups); or 2) when switching between one e-cigarette to another (2 groups). Participants in general agreed that regular, uninterrupted e-cigarette use required vigilance and planning. The e-cigarette devices would need constant monitoring. For example, the users needed to make sure, before they left home, that they had enough cartridges or e-liquid with them and the battery was fully charged. If in the middle of a party or a concert the battery died or they ran out of cartridges or e-juice, finding another e-cigarette was not as easy as finding another cigarette. Participants mentioned that the first few e-cigarette devices they experimented with were unsatisfying or of poor quality. As they switched between devices while trying to find the one they liked, in between, they continued to use cigarettes.

### Contexts of e-cigarette use

Table 3 lists the contexts of e-cigarette use that emerged across the 12 focus groups, supported by quotes that all authors agreed best exemplified the corresponding context. Overall, the contexts could be classified into three broad categories: activities that encourage e-cigarette use; places/situations that encourage e-cigarette use; and use of e-cigarettes as tobacco substitutes.

**Table 3:** Contexts of e-cigarette use

Contexts	Quotes
Activities	
Before/during work out or physical activity	“I use e-cig when I am engaging in more healthful activities.” (26 year old woman)
When working	“So it’s like when I’m working I can’t smoke a regular cigarette where I’m working at, so I can smoke this indoors. So when I’m not on break, and then when I’m on break, I switch.” (28 year old woman)
Places/situations	
At home/indoors,	“My family, they don’t smoke. So it’s basically if I want to smoke I have to smoke my e-cigarette and not a real cigarette, because I can’t smoke in the house. And of course I’m not going to go outside and smoke a real cigarette in the rain, because that’s not going to happen.” (28 year old man)
When alone	“Sharing e-cig is kinda gross. I mostly use e-cig when I am alone.” (22 year old man)
Inside a vehicle	“He went back to traditional cigarettes, and then he still uses his e-cig in his company van and stuff. Because he can’t smoke in there.” (26 year old woman)
When there is no time to take a shower after “smoking”	“When I don’t want to have to shower right after—e-cigarettes I like because I can smoke it, when I’m not stressed out it takes the edge off, and then I don’t stink.” (30 year old woman)
Tobacco substitute	
When cigarette is not available	“Yeah when I don’t have cigarettes, I use my e-cig.” (24 year old man)
When hookah is not available	“I like hokaah too. Because of the flavors. And e-cigs are like mini-hookah to me, without all the hassles of a hookah.” (23 year old woman)
When smoking is not allowed	“But cigarettes still, they fight the edge or what not. I’m smoking these, like the Logics. These Logic things are like ten bucks and last, it’s supposed to be like a carton… not a carton, but a pack of cigarettes, something like that. I’ll smoke one of those, keep it in my pocket at all times, you know what I mean? If I’m on the bus, chilling in the mall somewhere, as soon as I get out and there’s fresh air and I can smoke a cigarette, guarantee that cigarette is coming out of my pocket” (35 year old man)

#### Activities

Participants identified two activities that were conducive to e-cigarette use as opposed to cigarette smoking: 1) before or during work-out/physical activity (8 groups); and 2) when working (5 groups). Participants in general agreed that e-cigarette use was more conducive to physical activity. Specifically, participants mentioned that unlike cigarette smoking, e-cigarette use during or before physical activity did not affect their ability to perform. Cigarette smoking, on the other hand, made them feel “drained-out” and weak and adversely affected their breathing. Participants found it more convenient to use e-cigarettes while working. Unlike cigarette, e-cigarette use did not require them to take breaks to smoke. Co-workers were reported to be tolerant of their using e-cigarettes as they worked.

#### Places/situations

Dual users mentioned that certain places or situations were more conducive to e-cigarette use than cigarette smoking, specifically: 1) at home/indoors (11 groups); 2) when alone (4 groups); 3) inside a vehicle (9 groups); 4) when there is no time to take a shower after “smoking” (2 groups). Participants generally agreed that e-cigarette use was more suitable for indoor use: the vapor did not smell as bad and the risk of second hand exposure was perceived to be minimal. Participants mentioned that their non-smoking family members disapproved of their smoking cigarettes indoors. However, the same family members did not mind their e-cigarette use indoors. In addition, some of the parents among the participants reported that while they would not smoke cigarettes around their children, they freely used e-cigarettes around their children at home.

Dual users preferred to use e-cigarettes while in their private or work vehicle. They reasoned that smoking cigarettes in the car made the car smell bad. With e-cigarettes, one did not need to worry about the smell. The absence of cigarette-like smell also encouraged some dual users to use e-cigarettes when they did not have the time to take a shower after smoking. These dual users did not like the smell of cigarette on them. They were discreet smokers who smoked cigarettes only if they were not meeting anyone or had a chance to take a shower and change clothes after smoking. Some dual users believed smoking cigarettes or other tobacco products such as hookah to be a social activity. They perceived e-cigarette use to be essentially a solitary activity. An e-cigarette was thought to be more personal, not as easy to share as a cigarette or hookah.

#### Tobacco substitute

Across focus groups, 3 contexts emerged where e-cigarette was likely to be used by dual users as a substitute for cigarettes or hookah. These comprised use of e-cigarettes 1) when cigarette is not available; 2) when hookah is not available; and 3) when smoking is not allowed. Dual users who in general preferred cigarette or other form of tobacco smoking to e-cigarette use tended to use e-cigarette as a cigarette or tobacco substitute when cigarette or hookah was not available and/or when smoking cigarette or hookah was inconvenient or not permitted. These dual users used e-cigarettes to overcome craving temporarily until they got the opportunity to smoke tobacco. In the case of hookah users, however, e-cigarettes were mentioned to provide a much more convenient alternative.

## Discussions

Based on the current findings, it appears that cigarette smokers switching over to e-cigarettes may continue to use cigarettes when facing strong craving or the need for strong stimulation, until they find an e-cigarette device that satisfies such needs. Dual users seem to prefer cigarettes over e-cigarettes in situations where the need for the physiological effects of nicotine is paramount. Cigarettes are known to deliver nicotine more efficiently into the human body than e-cigarettes [15, 16]. Nicotine is a stimulant that increases the arousal of the central nervous system (CNS) and the sympathetic nervous system (SNS). Also, nicotine is an addictive substance that produces reinforcing effects by enhancing dopamine release in the brain’s reward pathways [17]. Because of their better nicotine-delivery efficiency cigarettes may better satisfy smokers’ craving than e-cigarettes [18] and provide more immediate, intense stimulation. In addition, cigarettes’ better nicotine delivery efficiency may be why dual users prefer cigarettes over e-cigarettes to relieve stress, to enhance the reinforcing aftereffects of food, sex, and physical activity or to use as laxatives. Further, for some dual users the nicotine delivered through cigarettes appear to provide the right level of relaxation and stimulation, before going to bed and after waking up, respectively. Although it is not clear how the reinforcing effects of nicotine as a stimulant help smokers relax or alleviate stress, smokers commonly report smoking cigarettes as a means of coping with stress [19].

Social influence may be an important barrier for cigarette smokers trying to substitute cigarette smoking with e-cigarette use. Mere physical proximity to individuals who possess or are smoking cigarettes may strongly encourage cigarette smoking among dual users. In addition, in social contexts where dual users are actively interacting with smokers, they may be directly or indirectly pressured into smoking cigarettes.

Dual users may show a variety of cigarette use patterns. Some dual users may use e-cigarettes throughout the week and use cigarettes on the weekend only; when they are likely to be partying with friends—possibly other smokers—and drinking alcohol. Other dual users may use e-cigarettes continuously for months and choose to use cigarettes on occasions that they consider to be special (e.g., New Year, birthday). Yet others seem to use cigarettes more frequently but secretly: they may smoke cigarettes in private when they can keep the smoking discreet but when interactions with people are imminent they may prefer to use e-cigarettes.

Use of addictive substances such as alcohol, caffeine, and marijuana may encourage cigarette smoking among dual users. Co-use of cigarette and alcohol, cigarette and marijuana, and cigarette and caffeine is widely prevalent [20, 21]. Cigarette smoking is believed to enhance the reinforcing effects of alcohol, marijuana or caffeine [21–23]. The stimulating effects of nicotine perhaps contribute to the increased subjective reinforcements associated with co-use. Thus, skills managing other substance use may matter greatly while trying to substitute cigarette smoking with e-cigarette use. In general, smokers attempting to quit smoking with the help of e-cigarettes may benefit from cognitive-behavioral skills training. For example, planning and vigilance seem important for regular, uninterrupted e-cigarette use. When e-cigarette users feel the urge to vape and are unable to use their e-cigarette devices for one reason or another, cigarettes serve as an easy substitute. If e-cigarette users are trained to manage their e-cigarette devices they may be more likely to have the products available to them when needed. This study did not probe participants’ views on the use of conventional Nicotine Replacement Therapy (NRT) products as substitutes of e-cigarettes when e-cigarettes are not available. It may be of interest to future research to determine e-cigarette users’ receptivity to conventional NRT products as substitutes for e-cigarettes when e-cigarettes are not available.

An important point to note is that not all dual users are cigarette smokers using e-cigarettes to quit smoking. There are dual users who use e-cigarettes opportunistically as cigarette substitutes. Contexts of e-cigarette use highlighted by the current findings elucidate such opportunistic use of e-cigarettes. E-cigarettes are a more convenient and discreet alternative to cigarettes for indoor use and in situations where tobacco smoking is either illegal or socially indiscreet. E-cigarette vapor does not smell as harsh as cigarette smoke and does not linger in the surrounding for long. Moreover, e-cigarette vapor is generally perceived to be less harmful than cigarette smoke [24] and to involve lower second-hand exposure risks [25].

An interesting finding of the current study is the use e-cigarettes as a hookah substitute. The smoke and vapor delivered by hookah and e-cigarettes, respectively, are both usually flavored. However, it takes time to set up a hookah. For example, the hookah has to be assembled and the coal has to be lit properly. Besides, for young adults, hookah smoking is a social activity; engaged in as a group. Participants indicated that hookah smokers preferred using e-cigarettes when they did not have time to set up a hookah or when they were alone and felt the urge to smoke.

The current findings have important implications for future research. Clearly, future research needs to determine what proportion of smokers who try e-cigarettes continue to use e-cigarettes to control versus aid their smoking behavior. Next, patterns and contexts of dual use needs to empirically studied through daily assessments in dual users’ natural environments. Such research would give a clearer picture of how different types of dual users use cigarettes and e-cigarettes. Such research would also help clarify the relative importance of different contexts in hindering smoking cessation or aiding continued cigarette smoking.

### Limitations

There are limitations to this study that need to be considered. One of the limitations is that participants across focus groups represented different types of duals users. For example, some participants were former dual users whereas others were current dual users. Some dual users were trying to use e-cigarettes to quit or reduce cigarette smoking whereas others were confirmed cigarette smokers who used e-cigarettes opportunistically, for convenience (e.g., indoors) or to deal with withdrawal and craving in situations where smoking cigarettes was not possible. Further, there might have been dual users in the sample who had previously quit smoking but had relapsed because of e-cigarettes. We failed to identify those dual users. Identifying such dual users would better elucidate contexts of cigarette and e-cigarettes use among relapsed ex-smokers. In general, it would be desirable to have the discussions separated by the types of dual users. Because the discussions were not thus separated, in-depth study of dual use contexts pertaining to each dual user type was not possible. This limited the scope of our findings. Secondly, we did not collect data on patterns of dual use. For example, based on the current data, a participant’s cigarette versus e-cigarette use patterns over a period of time cannot be ascertained. Because of this limitation, we were not able to identify how different contexts may influence frequency of cigarette and e-cigarette use. Thirdly, the present sample represented young adults (18–35 years old) only, because of which some of our findings may apply to young adults exclusively and not generalize to adolescents or older adults.

## Conclusions

Despite the limitations, this study is significant for advancing the current understanding of how dual use of cigarettes and e-cigarettes occurs. To our knowledge, this is the first study to attempt to elucidate the contexts of e-cigarette and cigarette use among dual users. The findings have important implications for application of e-cigarettes in smoking cessation or tobacco harm reduction. First, the results highlight the fact that cigarettes smokers trying to switch over to e-cigarette use may frequently give in to the triggers and cues that demand the more intense stimulating effects of cigarettes. Second, continued cigarette smoking in one’s social environment may contribute strongly to dual use. Smokers trying to switch over to e-cigarette use may be easily influenced into smoking cigarettes by friends and family members who smoke cigarettes. Third, smokers trying to replace cigarette smoking with e-cigarette use may need to vigilantly manage their e-cigarette devices in order to ensure that the devices are available when needed.

It is possible that effective application of e-cigarettes in smoking cessation and/or harm reduction may partly depend on improved technology on e-cigarettes’ part in delivering nicotine efficiently. However, more importantly, promotion of e-cigarette use in smoking cessation or harm reduction may need to be coupled with counseling focused on training smokers on how to manage e-cigarette devices and behaviors and situations associated with tobacco smoking. In addition, research is needed to understand the characteristics of dual users who are attempting to quit cigarette smoking versus those who are using e-cigarettes to aid smoking habit. The opportunistic use of e-cigarettes to facilitate continued use of cigarettes is obviously of concern.
